# Factors associated with disability among patients with leprosy: a scoping review

**DOI:** 10.1186/s12879-026-14255-w

**Published:** 2026-08-19

**Authors:** Clausewitz Welmatus Masala, Hartiah Haroen, Iqbal Pramukti

**Affiliations:** 1 Master of Nursing Program, Faculty of Nursing, Universitas Padjadjaran, Sumedang, West Java 45363 Indonesia; 2 Nursing Study Program, Faculty of Medicine, Universitas Kristen Indonesia, East Jakarta, Jakarta, 13630 Indonesia; 3 Department of Nursing Community, Faculty of Nursing, Universitas Padjadjaran, Sumedang, West Java 45363 Indonesia

**Keywords:** Disability, *Morbus Hansen*, Patients with leprosy

## Abstract

Hansen’s disease, often known as leprosy, is a chronic infectious disease caused by *Mycobacterium leprae* that mostly affects peripheral nerves, the skin and mucous membranes. Notwithstanding improvements in diagnosis and treatment options, many patients with leprosy remain severely disabled, which negatively impacts their quality of life and social interaction. Determining the factors associated with the development of disability in patients with leprosy is essential for developing successful therapeutic plans. For the purpose of this review, disability is defined according to the WHO grading system as any impairment of the eyes, hands, or feet caused by leprosy (Grade 0–2). This review aimed to identify the factors associated with disability among patients with leprosy. The guiding research question was: What are the factors associated with the occurrence of disability in patients with leprosy?. This study employed a scoping review following the guidelines provided in the PRISMA Extension for Scoping Reviews (PRISMA-ScR). Literature searches were conducted using two databases and search engines, namely PubMed, Scopus, and Google Scholar. Articles included in the review were published in English and Indonesian between 2019 and 2023, a period selected to capture the most recent evidence reflecting contemporary clinical practices and disability assessment approaches in leprosy management. Eligible studies were selected and analyzed after approval. The quality of the evidence was assessed using the Joanna Briggs Institute (JBI) criteria. Data extraction was carried out using a standardized form that captured information on study characteristics, participant demographics, and key findings related to factors influencing the disability among patients with leprosy. This review analyzed 10 articles, identifying 12 factors that influence the disability in patients with leprosy. These factors include the type of leprosy, duration of the disease, leprosy reactions, the number of affected nerves, delays in treatment or diagnosis, socioeconomic status, gender, occupation, education level, treatment adherence, self-care, and social support. These influencing factors were categorized into three groups: demographic factors, internal factors, and external factors. These findings highlight the complex interplay of various factors contributing to disability in patients with leprosy, emphasizing the need for targeted interventions that address both individual and systemic issues to improve patient outcomes.

## Introduction

Leprosy is an infectious disease that causes complex and multidimesional problems. These problems are not only limited to medical aspects but also extend to social, economic, cultural, and public health dimensions. To date, leprosy remains a source of fear among communities, families, and even some healthcare workers [1, 2]. This is largely due to inadequate knowledge and persistent misconceptions about the disease and its potential to cause disability [3, 4]. Disability in leprosy primarily result from impaired peripheral nerve function, affecting various parts of the body such as the eyes, hands, and feet [5, 6]. The risk of disability increases with delays between early symptom recognition and treatment initiation, as progressive nerve damage may lead to permanent impairment [3, 7–9].

Leprosy is classified as one of the Neglected Tropical Diseases (NTDs) and remains prevalent in many countries worldwide. According to the World Health Organization (WHO) leprosy continues to be a significant global public health concern. The most recent Global Leprosy Update reported that in 2023, a total of 182.815 new leprosy cases were detected globally, showing an increasing trend compared to previous years [10]. The majority of cases were reported from the South-East Asia Region, followed by the African and Americas region, indicating ongoing transmission in endemic areas [10].

In Indonesia, leprosy remains a major public health issue with a consistently high number of new cases. According to the WHO report (2024), Indonesia reported 10,976 new cases in 2021, 12,441 cases in 2022, and 14,376 cases in 2023 [11]. This upward trend indicates ongoing transmission and challenges in early detection and control efforts [11]. At the subnational level, local data still show a considerable burden of disability among newly detected cases. For example, a report from the Cirebon District Health Office in 2022 indicated that among newly detected cases, a substantial proportion were classified as *multibacillary* (MB), with a notable percentage already presenting with grade 2 disability [12]. These findings suggest that delayed diagnosis and disease progression remain critical issues, contributing to the persistence of disability among patients with leprosy.

Although numerous studies have explored leprosy and its related disabilities, gaps remain in understanding the specific factors contributing to disability among affected individuals. These factors may include delayed diagnosis [13, 14], limited access to healthcare services [15, 16], social stigma [17, 18], poverty [19, 20], and cultural influences on healthcare-seeking behavior [21, 22].

This study employs a scoping review approach to systematically map the existing literature on factors associated with disability among patients with leprosy and to identify research gaps that have not been extensively explored. This approach was chosen to provide a comprehensive understanding of the relationship between clinical, social, economic, and cultural determinants of disability in leprosy [23]. In this review, disability is defined according to the WHO leprosy disability grading system as any impairment of the eyes, hands, or feet attributable to leprosy, classified as Grade 0 (no disability), Grade 1 (decreased sensation without visible deformity), or Grade 2 (visible deformity or damage). This operational definition guided the selection and analysis of included studies. To address this issue, a study was conducted with the following research question: What are the factors associated with the occurrence of disability among patients with leprosy? The objective was to perform a comprehensive scoping review of the literature on factors that contribute to disability among patients with leprosy.

## Methods

### Design and sample

This scoping review was carried out based on the framework proposed by Arksey and O’Malley (2005), with modifications and improvements introduced by Levac, Colquhoun, and O’Brien (2010) [24]. It is important to clarify the methodological distinctions between review types. A systematic review rigorously synthesizes evidence to answer a specifics research question using strict eligibility criteria, risk-of-bias assessment, and often quantitative pooling. A meta-analysis a statistical method used within a systematic review to quantitatively combine effect estimates across studies. In contrast, a scoping review is designed to map the breadth of a topic, identify key concepts, and characterize the available evidence without necessarily appraising study quality or drawing definitive conclusions. This review employs the scoping review methodology because the factors associated with leprosy-related disability span heterogeneous domains (clinical, behavioural, socioeconomic) and multiple study designs, making it more appropriate to map and characterize this evidence than to synthesize it through a systematic review. Although only 10 articles met the final eligibility criteria, this reflects the narrow focus of the search parameters (published 2019–2023, English or Indonesian language), and the purpose remains exploratory mapping rather than exhaustive synthesis. The scoping review method offers a flexible approach to investigating topics that are rapidly evolving [25]. This is particularly applicable to the topic of disability in leprosy, as emerging evidence continues to highlight the interaction between clinical progression, social determinants, and health system factors, which are not yet fully synthesized in the literature [25]. In the context of disability among patients with leprosy, where various interconnected factors play a role, this exploratory approach aids in uncovering aspects that may have been overlooked in existing literature.

The methodology consists of five key stages: formulating the research question, identifying relevant studies, selecting studies, mapping the data, and gathering, summarizing, and presenting the findings [25]. In this literature review, the PRISMA Extension for Scoping Reviews (PRISMA-ScR) guidelines were utilized to identify factors linked to the occurrence of disabilities in patients with leprosy. This review was not registered in a prospective protocol database. As a graduate-level research project conducted without external funding, prospective registration (e.g., in OSF or PROSPERO, which does not accept scoping reviews) was not feasible. The authors acknowledge this as a limitation and recommend that future reviews of this topic be prospectively registered to enhance transparency.

### Eligibility criteria

The articles included in this review were chosen based on the PRISMA Extension for Scoping Reviews (PRISMA-ScR) guidelines [26]. This article uses the PCC (Population, Concept, and Context) approach to formulate the research questions and eligibility criteria. The approach includes: P (Population) referring to patients with leprosy, C (Concept) encompassing risk factors, predictors, and determinants related to leprosy, and C (Context) focusing on disability experienced by patients with leprosy.

Inclusion criteria included: (a) Subjects related to patients with leprosy among disabilities; (b) Full-text articles; (c) The languages used include Indonesian and English, the use of both languages in scientific articles aims to enhance accessibility for local and international audiences; (d) Publication within the last 5 years (2019–2023). The exclusion criteria include: (1) conference papers, (2) non-peer-reviewd articles, and (3) articles with only abstracts available.

### Data collection and analysis

#### Search strategy

Article retrieval uses two databases PubMed and Scopus and one search engine, Google Scholar. These databases were selected because PubMed provides comprehensive biomedical and clinical literature, Scopus offers broad multidisciplinary coverage with high-quality indexed journals, and Google Scholar allows inclusion of grey literature and additional relevant studies not indexed in traditional databases. Article search was conducted in March 2024. Keywords use two languages, namely Indonesian for Google scholar and English Keywords for PubMed and Scopus. Keywords “Leprosy” OR “*Morbus hansen*” AND “Disability” AND “Factor” OR “Predictor” OR “Determinant” (see Table 1).

**Table 1:** Search strategy

Database	Search Strategy
PubMed	(“Leprosy“[MeSH] OR “Leprosy“[Title/Abstract] OR “Morbus Hansen“[Title/Abstract]) AND (“Disability“[MeSH] OR “Disability“[Title/Abstract]) AND (“Factor“[Title/Abstract] OR “Predictor“[Title/Abstract] OR “Determinant“[Title/Abstract])
Scopus	(TITLE-ABS-KEY(“Leprosy”) OR TITLE-ABS-KEY(“Morbus Hansen”)) AND (TITLE-ABS-KEY(“Disability”)) AND (TITLE-ABS-KEY(“Factor”) OR TITLE-ABS-KEY(“Predictor”) OR TITLE-ABS-KEY(“Determinant”))
Google Scholar	(“Leprosy” OR “Morbus Hansen”) AND “Disability” AND (“Factor” OR “Predictor” OR “Determinant”)

### Study selection and quality appraisal

All authors were responsible for selecting studies that fulfilled the eligibility requirements. Using reference management software (Mendeley), the authors screened for duplicates during the initial selection phase. Subsequently, they evaluated the title, abstract, and full text for relevance to the research topic, establishing inclusion and exclusion criteria. In the final stage, two authors conducted a critical review of each study using the JBI Critical Appraisal Tools (Joanna Briggs Institute, available at: https://jbi.global/). A third author then conducted a final check.

A total of 932 articles were identified from the search results and subsequently filtered using predetermined criteria. First, duplicate articles were removed, leaving 908 articles for further screening. These articles were then assessed based on their titles and abstracts, resulting in the exclusion of 829 articles that were deemed irrelevant to the study focus. The remaining 79 articles underwent a more comprehensive evaluation based on inclusion criteria, such as population, intervention, and publication language. From this stage, 69 articles were excluded due to specific reasons, including studies on leprosy patients with disabilities not caused by leprosy (34 articles), articles not published in English or Indonesian (6 articles), and articles with outcomes that did not align with the research analysis (29 articles).

**Fig. 1:**
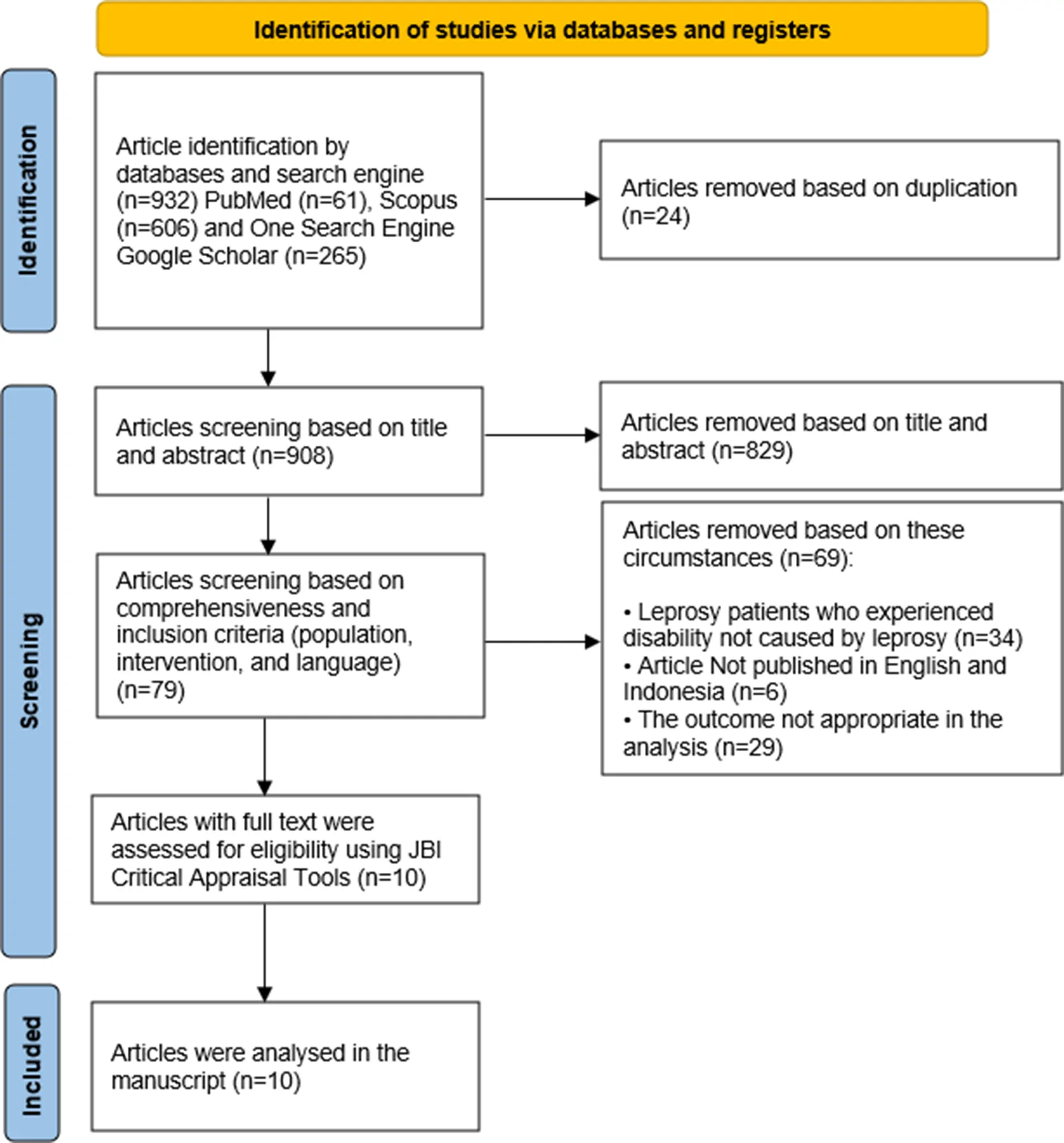
PRISMA flow diagram. Notes: PRISMA Flow Diagram adapted from Page MJ, McKenzie JE, Bossuyt PM, et al. The PRISMA 2020 statement: an updated guideline for reporting systematic reviews. *BMJ*. 2021;372:n71. Creative Commons [26]

Following this screening process, 10 articles met the initial criteria and were further assessed by two reviewers using the JBI Critical Appraisal Tools (see Table 2). Articles that achieved a score of more than 50% were considered eligible for review. The complete search flow is illustrated in Fig. 1, Study Search Flow Chart.

**Table 2:** Critical appraisal results

Study	Design	JBI Critical Appraisal Tool
Kentari et al., 2019 [27]	Cross Sectional	8/8 (100%)
Chen et al., 2023 [28]	Case Series	10/10 (100%)
Li et al., 2021 [29]	Case Series	10/10 (100%)
Arun & Maulana, 2019 [30]	Case Control	8/10 (80%)
Tuturop, Adimuntja, & Borlyin, 2022 [31]	Case Control	9/10 (90%)
Maulina et al., 2023 [32]	Cross Sectional	7/8 (87.5%)
Menaldi et al., 2022 [33]	Cross Sectional	8/8 (100%)
Widya et al., 2019 [34]	Cross Sectional	7/8 (87.5%)
Geani et al., 2022 [35]	Cross Sectional	9/10 (90%)
Srinivas et al., 2019 [36]	Case Control	8/8 (100%)

### Data extraction and analysis

In this review, the data extracted from the studies are analyzed through tables, which provide a detailed description of all findings related to the topic under discussion. The data extraction for this review was conducted by the first author and verified by the other authors. The information presented in the extraction table includes details on study design, such as the country, research design, research subjects, instruments, and results.

The data were analyzed using thematic analysis. The process of analysis began by identifying and presenting the collected data in a tabular format based on the reviewed articles. Once the data was gathered, the author examined and explained each finding in relation to the factors that may influence the disability. The identification of specific predictor themes or categories followed a systematic analytical approach. Initially, the extracted predictor data were examined to recognize emerging patterns or trends to determine the categories of disability predictors (see to Table 3). Subsequently, similar categories were consolidated into broader themes. Each stage of this process was discussed collaboratively by the research team to ensure the themes were consistent, transparent, and aligned with the research goals.

Specifically, themes were developed inductively: the first author independently coded the extracted predictor data from each study into preliminary categories based on the wording and context used by the original authors, after which the second author independently reviewed the same extraction table and proposed an initial thematic grouping. The two sets of categories were then compared, and any discrepancies in labelling or grouping were resolved through discussion between the two coders until consensus was reached; where disagreement persisted, the third author acted as an arbiter to reach a final decision. Coding consistency across reviewers was further checked by cross-referencing each factor against the original article to confirm that the assigned theme (demographic, internal, or external) accurately reflected how the factor was measured and reported in the source study. This iterative, consensus-based process was used instead of a formal inter-rater reliability statistic, consistent with the descriptive, exploratory nature of thematic analysis in scoping reviews.

## Results

This study provides a comprehensive examination of various factors associated with leprosy and their potential contribution to disability among affected patients, encompassing demographic, internal, and external dimensions (see Fig. 1 and Table 4). Importantly, this review also identifies several research gaps, particularly related to inconsistencies in demographic findings and limited integration between clinical and socio-environmental factors.

Internal factors include individual clinical characteristics that are directly related to the disease condition. The type of MB leprosy has been identified as an important clinical factor associated with greater disease severity and, indirectly, with an increased risk of disability [28, 30–35]. Of these [31], examined factors associated with leprosy occurrence rather than disability directly, so its contribution here is treated as indirect, contextual evidence rather than a direct disability finding. Patients with MB leprosy tend to experience more extensive bacterial load and nerve involvement compared to those with *paucibacillary* (PB) leprosy. In addition, the duration of leprosy is an important consideration, as prolonged disease may lead to progressive tissue and nerve damage, thereby increasing the likelihood of disability [28, 32, 35]. It should be noted that this association was not observed consistently: [32], a smaller cross-sectional study (*n* = 47), found no significant relationship between disease duration and disability grade (*p* = 0.982), so disease duration is best regarded as a plausible but unconfirmed factor rather than an established predictor. The characteristics of the included studies reflect a diverse population of patients with leprosy, allowing for a broader understanding of how these factors may influence disease progression and subsequent disability outcomes.

Leprosy reactions experienced by patients also play a crucial role in disease progression and the potential development of disability [35, 36]. These immunological responses to *Mycobacterium leprae* infection can trigger acute inflammation, leading to nerve damage and functional impairment if not promptly managed. Furthermore, the number of nerves affected represents another important internal factor, as greater nerve involvement increases the likelihood of sensory and motor impairments that may result in disability [28, 35, 36]. The more nerves that are affected, the more likely the patient is to experience sensory and motor impairments that can lead to disability.

External factors, such as delays in diagnosis or initiation of treatment, are also critical determinants. Delayed case detection and treatment initiation can contribute to more advanced disease progression and may increase the risk of irreversible complications, including disability [27, 28, 30, 34, 36]. These findings highlight the importance of early detection and timely intervention in preventing long-term consequences of the disease.

Demographic and socio-economic factors further influence both disease occurrence and its progression. Socio-economic conditions may affect access to healthcare services and treatment adherence, thereby indirectly contributing to the risk of disability [29, 33]. In addition, factors such as gender [28–36], occupation [28–30, 33, 34, 36], education level [28–34, 36], medication adherence [27–31, 35], self-care practices [27, 36] and social support from family, peers, and the community [27, 30, 31, 36] have been reported to influence leprosy occurrence and management. Although these factors are not always directly linked to disability outcomes, they may indirectly affect the risk of disability through mechanisms such as delayed diagnosis, poor treatment adherence, and limited access to care.

The findings across studies showed both consistent and inconsistent results. Clinical factors such as MB type and diagnostic delay consistently demonstrated a positive association with disability across multiple studies [28, 30, 36]. Similarly, delayed diagnosis and prolonged disease duration were associated with increased disability risk [35], although not consistently across all studies [32]. In contrast, demographic factors revealed substantial inconsistencies across studies, representing a key research gap. Age and education were associated with disability in some studies [33, 35] but not in others [32], indicating variability across populations and study designs. Gender, in particular, was mostly reported as not significantly associated with disability [32, 35], although it was still described as a potential influencing factor in several studies [28, 33].

Behavioral and external factors, including self-care, treatment adherence, and delayed diagnosis, were more consistently associated with disability outcomes [27, 30, 36]. However, limited studies have explored how these behavioral factors interact with socio-cultural contexts, highlighting another important gap in the literature. Overall, these findings suggest that while clinical predictors are relatively well established, there remains limited evidence integrating clinical, behavioral, and socio-economic factors into a comprehensive explanatory model of disability in leprosy.

**Table 3:** Data extraction results: factors associated of disability among patients leprosy

No	Article Title, Author &Year	Research Design and Analysis	Research Subject	Data Collection Instruments	Result	Factors Associated with Disability	Key Findings
1	Kentari et al., 2019 [27]	Cross Sectional and logistic regression tests	107 Leprosy patients at the Outpatient Hospital of Kediri Leprosy Hospital.	A questionnaire	Self-care has a greater impact than MDT treatment regularity on hand (*p* = 0.002) and leg (*p* = 0.000) disabilities in leprosy patients. Family support primarily influences MDT treatment regularity, while healthcare workers play a more significant role in promoting self-care. Self-care has a stronger effect on hand and foot disabilities compared to MDT treatment regularity.	Self-care, MDT adherence	Self-care showed a stronger association with disability than MDT adherence
2	Chen et al., 2023 [28]	Case series and Descriptive statistical analysis	Patients with clinical manifestations and positive laboratory tests for leprosy	Data on newly diagnosed cases of leprosy were collected from the leprosy management information system in China	The G2D rate among migrant, resident, and total leprosy patients was 17.5%, 18.7%, and 18.4%, respectively. From 2001 to 2021, the total G2D rate significantly increased from 18.0% to 25.7% (2.5% annual change). Delayed discovery time and nerve damage at diagnosis were major risk factors, with higher odds among residents (OR = 4.99 and 21.28) than migrants (OR = 2.57 and 9.40).	Delay, nerve damage	Delayed diagnosis and nerve damage increased risk of disability
3	Li et al., 2021 [29]	Case series with chi-square testing and logistic regression analysis.	From 1986 to 2015, a total of 3,376 leprosy cases were detected in Wenshan. The	Data in Wenshan, China were collected from the Wenshan Institute of Dermatology (1986–2015); data in Nepal were obtained from the Leprosy Control Division, Department of Health Services, Nepal (2011 to 2015); and data from Indonesia, India, and Brazil were collected from WHO records.	Geographic latitude, socio-economic conditions, and healthcare quality significantly influence leprosy incidence. The introduction of MDT has effectively reduced its prevalence globally. Wenshan (China), Nepal, and similar regions share socio-cultural, geographical, environmental, and economic factors that contribute to leprosy endemics.	Socio-economic, healthcare access	Influenced leprosy incidence (indirectly related to disability)
4	Arun & Maulana, 2019 [30]	*Case Control* and uji *chi square*	40 Leprosy patients recorded in medical records in the Pekalongan City health centre working area.	Kuesioner	The study found a significant relationship between early screening (*p* = 0.000; OR = 12.000; 95% CI = 2.700–53.330) and treatment adherence (*p* = 0.003; OR = 8.500; 95% CI = 1.861–38.817) with disability in leprosy patients, while family support (*p* = 0.429; OR = 1.889; 95% CI = 0.385–9.271) showed no significant association.	Early detection, treatment adherence	Both factors significantly associated with disability
5	Tuturop, Adimuntja, & Borlyin, 2022 [31]	*Case control study* and uji *Chi-square*	25 leprosy patients visited primary health care unit Kotaraja in 2021	*Informed Consent*, questionnaires and stationery	The study results indicate that education level (OR = 11.1, 95% CI: 2.86–43.46), contact history (OR = 13.5, 95% CI: 1.55–117.13), treatment adherence (OR = 6.68, 95% CI: 1.76–25.24), and family support (OR = 5.63, 95% CI: 1.64–19.23) are risk factors for leprosy. However, age (OR = 0.47, 95% CI: 0.41–5.65) and gender (OR = 2.11, 95% CI: 0.62–7.13) are not associated with leprosy occurrence.	Education, contact, adherence, family support	Associated with leprosy occurrence (indirectly related to disability)
6	Maulina et al., 2023 [32]	*Cross sectional and Kolmogorov-Smirnov test with a significance decision of 5% (α = 0.05).*	47 leprosy patients in primary health care unit in the Lhokseumawe City working area in 2016-2020	Treatment monitoring book and disability book of registered leprosy patients from 2016-2020	The study found that most respondents were in the productive age group (74.5%), male (61.7%), and had low (46.8%) to moderate (46.8%) education levels. Statistical analysis using the Kolmogorov-Smirnov test (α = 0.05) showed no significant relationship between age (*p*=1.000), gender (*p*=1.000), education level (*p*=1.000), leprosy type (*p*=0.905), and disease duration (*p*=0.982) with the degree of leprosy disability. Therefore, it can be concluded that these factors are not associated with the level of disability in leprosy patients at health centers in Lhokseumawe.	Age, gender, education, type, duration	No significant association with disability
7	Menaldi et al., 2022 [33]	A cross-sectional study with Kruskal-Wallis and Mann-Whitney test	267 retrospectively-diagnosed cases of leprosy	The Screening of Activity Limitation and Safety Awareness (SALSA) scale was used tomeasure functional activity limitation, which comprises five domains: vision, mobility, selfcare, work with hands, and dexterity.	The participants had a mean age of 51.89±13.66 years, with most being men (62.5%), uneducated (48.3%), and classified as WHO disability grade 2 for hands (66.3%) and feet (68.2%). The SALSA Scale assessment showed 28.5% had no limitation, while 43.8% had mild, 13.5% moderate, 9.4% severe, and 4.9% extreme limitations. Significant differences in SALSA scores were found based on age (*p* = 0.014), education (*p* = 0.005), occupation (*p* < 0.001), and disability grade (*p* < 0.001). Multivariate analysis identified foot disability grading as the strongest predictor (score = 0.31, *p* < 0.001), followed by occupation, eye disability grading, and age. Functional activity limitation was significantly associated with unemployment (OR = 2.59).	Age, education, occupation	Significantly associated with functional disability
8	Widya et al., 2019 [34]	*Cross sectional*	144 leprosy patients at Pemalang District Health Centre in 2017	Interviews and secondary data	The analysis shows a disability proportion of 27.9%. Respondents over 14 years old accounted for 29.0%, males 28.7%, those with low education levels 34.9%, and farmers 30.42%. MB-type leprosy was found in 29.0%, low knowledge about leprosy in 33.7%, and passive case detection in 28.1%. Disability is higher among respondents over 14 years old, males, those with low education and knowledge, farmers, MB-type leprosy patients, and cases detected passively. Further research with analytical methods is recommended.	Age, gender, education, MB type, knowledge	Higher disability observed in these groups (descriptive)
9	Geani et al., 2022 [35]	A cross-sectional retrospective analytical study	275 leprosy patients with disabilities	Medical record	This study identified 275 leprosy patients with disabilities, including 76 (27.6%) with grade-1 and 199 (72.4%) with grade-2 disabilities. Age (*p*=0.025), disease duration (*p*=0.001), and MDT history (*p*=0.001) were significantly associated with disability, while gender, bacterial index, leprosy type, and reaction showed no significant correlation.	Age, duration, MDT history	Significantly associated with disability
10	Srinivas et al., 2019 [36]	Case-control study	700 cases with disability (G2D or G1D) and 700 controls without disability (G0D)	Close-ended structured questionnaire by trained research staffs	A total of 1,400 new leprosy patients from five states were interviewed. The median patient delay was 8 months for G2D/G1D and 4 months for G0D, while the median HCP delay was 2 months and 1 month, respectively. The total median delay was significantly longer for G2D/G1D (14 months) than for G0D (6.2 months) (*p*<0.001). Patients with delays exceeding 3 months had 1.6 times higher odds of G2D/G1D at diagnosis, while an HCP delay of more than 1 month increased the odds by 1.4 times. Multi-bacillary leprosy was associated with nine times higher odds of G2D/G1D compared to pauci-bacillary leprosy.	Diagnostic delay, MB type	Strong association with disability risk

Abbreviations: G0D (Grade 0 disabilities), G1D (Grade 1 disabilities), G2D (Grade 2 disabilities), MDT (Multidrug therapy), SALSA (Screening of Activity Limitation and Safety Awareness), type-MB (type *Multi-Bacillary*)

## Discussion

This scoping review synthesizes evidence from ten studies published between 2019 and 2023 to characterize the factors associated with disability among patients with leprosy. Drawing directly from the included studies, the analysis identifies 12 factors organized into three domains: demographic, internal, and external. Rather than simply describing background information on each factor, this section discusses what the included studies specifically found and where the evidence agrees, conflicts, or remains limited. Regarding methodological quality, the JBI critical appraisal scores across included studies ranged from 80% to 100%. Cross-sectional studies (Kentari et al., [27]; Maulina et al., [32]; Menaldi et al., [33]; Widya et al., [34]; Geani et al., [35]) scored 87.5–100%, reflecting adequate reporting of study design and objectives but noting that cross-sectional designs limit causal inference. Case-control studies (Arun & Maulana, [30]; Tuturop et al., [31]; Srinivas et al., [36]) scored 80–90%, with the largest study (Srinivas et al., *n* = 1400) providing the most statistically robust findings on diagnostic delay and MB type as disability predictors. The two case series (Chen et al., [28]; Li et al., [29]) each achieved 100% on the JBI checklist; however, both present inherent limitations in inferring causal associations due to the absence of a control group. These quality considerations inform how confidently each factor can be interpreted. The analysis was organised into three interrelated factors: demographic, internal and external (see Table 4). Importantly, two included studies (Li et al. [29] and Tuturop et al. [31]) examined leprosy incidence rather than disability directly; their findings are discussed as contextually relevant but are noted as indirect evidence with respect to disability outcomes.

An analysis of 10 studies revealed that several factors contribute to disability in patients with leprosy. These factors include the type of leprosy MB, duration of the disease, leprosy reactions, the number of nerves involved, delayed diagnosis or treatment, socioeconomic status, gender, occupation, education level, adherence to medication, self-care practices, and social support. Notably, seven of the ten studies were conducted in Indonesia, which is a high-burden setting with specific cultural, healthcare, and socioeconomic characteristics. This geographic concentration should be considered when interpreting findings, as the associations observed may not generalize to other endemic settings such as India, Brazil, or sub-Saharan Africa, where disease dynamics and health system factors differ substantially.

**Table 4:** Factors influencing the incidence of disability

Factors	Theme	Study
Demographic factors	Social economy	[29, 33]
	Gender	[28, 30–36]
	Occupation	[28, 30, 33, 34, 36]
	Education level	[28, 30–34, 36]
Internal factors	Type of MB leprosy	[28, 30–35]
	Duration of leprosy	[28, 32, 35]
	Number of Nerves affected	[28, 35]
	Reaction to leprosy	[28, 35]
External factors	Delay in Treatment / Diagnosis	[27, 28, 30, 34]
	Treatment Regularity	[27–31, 35]
	Self-care	[27, 36]
	Social Support	[27, 30, 31, 36]

Note: References [29] and [31] (Li et al., 2021 and Tuturop et al., 2022) examined factors associated with leprosy occurrence/incidence rather than disability directly. Their citation in this table reflects contextually relevant, indirect evidence and should not be interpreted as direct evidence for disability outcome

### Demographic factors

1. **Socio-Economic**

People with leprosy often face complex socioeconomic challenges. Stigma and discrimination against the disease can lead to social isolation, difficulty finding employment, and even loss of income [37]. The physical limitations caused by the disease may also limit their access to economic opportunities [38]. With these limitations, leprosy may face difficulties in accessing adequate health services and education, as well as limited opportunities to develop the skills necessary to enter the job market. These complex socio-economic impacts require a holistic and inclusive approach to economically and socially empower leprosy sufferers, including efforts to reduce stigma, improve access to health and education services, and provide skills training and inclusive employment opportunities.

2) **Gender**

Leprosy, an infectious disease that has long haunted humanity, often has a significant impact on sufferers, especially in terms of disability. One important aspect to consider in this context is gender differences. Research shows that female leprosy sufferers tend to have higher rates of disability compared to males, possibly due to complex biological, social and economic factors [39–43]. These differences can be reflected in patterns of transmission, diagnosis and access to adequate care. This broader literature should, however, be distinguished from what the ten included studies themselves showed: within this review, gender was reported as not significantly associated with disability in [32] and [35], while it was described as a potentially influencing factor in only [28] and [33]. Gender should therefore be treated as a factor with mixed and currently inconclusive evidence within this body of literature, rather than an established predictor of leprosy-related disability. Thus an in-depth understanding of the role of gender in the experience of disability in people with leprosy is critical to designing effective and equitable interventions for all individuals affected by the disease.

3) **Occupation**

The disability in people with leprosy often presents significant challenges in terms of employment. Although modern medicine has reduced the rate of disability caused by the disease, social stigma and discrimination against leprosy sufferers are still major barriers to finding and maintaining employment [44]. Many companies and individuals are still affected by the negative stereotypes associated with leprosy, making it difficult for sufferers to be accepted in the workplace [45]. In addition, the physical complications that may arise from the disease, such as nerve damage or loss of limbs, may limit one’s ability to perform job tasks effectively [46]. Therefore, it is important to promote inclusion and awareness of the rights of people with disabilities in the world of work, as well as provide adequate accessibility so that people with leprosy can develop their full potential.

Within the included studies, occupation showed a more consistent statistical association than gender or education, being significantly linked to disability or functional limitation in [33] (a moderately sized cross-sectional study, *n* = 267) and [34], although it was not examined as a distinct variable in several of the other studies. Given the limited number of studies that tested occupation directly, this factor should be interpreted as a preliminary rather than a firmly established predictor.

4) **Educational Level**

The disability in leprosy sufferers often has a significant impact on their level of education. Due to strong social stigma, many leprosy sufferers experience discrimination and denial of access to formal education [47, 48]. This leads to low levels of literacy and formal education among them, worsening their ability to integrate into society and the labor market [49]. People with leprosy often face physical and psychological challenges that can hinder their participation in the education process, be it access to school or consistent attendance. Therefore, a holistic approach that takes into account the physical, psychological and social needs of people with leprosy is needed to ensure equal access to education and minimize the educational disparities caused by this disability.

As with gender, the association between education level and disability was inconsistent across the included studies: [33] and [34] reported a significant relationship, whereas [32], despite a small sample size (*n* = 47), found no significant association (*p* = 1.000). Given this inconsistency and the small sample sizes involved in several of these studies, education level should currently be regarded as a possible rather than a confirmed predictor of disability in this population.

### Internal factors

1. **Type Leprosy**

Leprosy, also known as Hansen’s disease, is a chronic infectious disease that can cause damage to the skin, peripheral nerves, and internal organs. There are two main types of leprosy that affect humans: lepromatous (*multibacillary*) and tuberculoid (*paucibacillary*) [50, 51]. The lepromatous type is characterized by extensive and more severe infection, resulting in widespread skin lesions and significant nerve damage [52–54]. On the other hand, tuberculoid types tend to show more localized skin lesions and a better immune response to the *Mycobacterium leprae* bacteria [52, 55]. Both types of leprosy can cause varying degrees of disability, ranging from peripheral nerve damage to limb loss, which can have a serious impact on the quality of life of sufferers.

2) **Duration of Leprosy**

The disability in people with leprosy, especially those with long-standing conditions, is a complex phenomenon that requires in-depth understanding from various perspectives, both medical and social. At the medical level, prolonged leprosy can result in significant peripheral nerve damage, leading to loss of sensory and motor function that can cause permanent disability [56, 57]. In addition, social aspects also play a role in amplifying the negative impact, with stigma and discrimination against leprosy sufferers often hampering their access to health services, education, and employment opportunities, leaving them socially and economically marginalized [58–60]. Therefore, holistic and sustainable treatments need to be implemented to address the challenges faced by long-standing leprosy sufferers, taking into account both medical and social aspects to ensure a better quality of life for them.

3) **Number of Nerve Affected**

In people with leprosy, the disability is often related to the number of nerves affected. The disease can cause damage to the peripheral nerves, which control sensation and movement in certain parts of the body [61, 62]. As a result, people with leprosy often experience a loss of sensation in the skin, especially in areas affected by the affected nerves [56]. This can lead to wounds or injuries that go unnoticed and have the potential to develop into serious infections. In addition, nerve damage can also lead to muscle weakness and loss of ability to move limbs normally. As such, the number of nerves affected in a case of leprosy greatly affects the severity of the disability experienced by the sufferer.

4) **Leprosy Reaction**

Leprosy reaction is one of the most common clinical manifestations of leprosy, a chronic disease caused by the bacterium *Mycobacterium leprae*. This reaction is an exaggerated immune response to the bacterial infection, which can result in inflammation and tissue damage [52, 63]. There are two main types of leprosy reactions: type 1 or reversal reactions, which are characterized by inflammation of the skin and peripheral nerves, and type 2 or *erythema nodosum leprosum* (ENL) reactions, which are characterized by subcutaneous nodules and systemic manifestations such as fever and joint pain [63–65]. Both types of reactions can cause significant nerve damage, which in turn can lead to disability in people with leprosy. Prompt and appropriate treatment of leprosy reactions is crucial in preventing further damage and improving the quality of life for people with leprosy.

### External factors

1. **Delay in Treatment/Diagnose**

Delays in diagnosis and treatment of people with leprosy are often the main cause of significant disability. This is often triggered by the lack of public understanding of the early symptoms of leprosy, as well as the lack of accessibility to appropriate and quality health services [66–69]. Late diagnosis results in a delay in necessary treatment, allowing the disease to progress to a more severe stage, causing irreversible nerve damage and resulting in permanent disability [56, 70, 71]. Social, economic and cultural factors exacerbate this situation, with some marginalized communities experiencing difficulties in accessing appropriate health services. Therefore, a better understanding of leprosy, improved accessibility to health services, and wider socialization of the importance of early detection are crucial in reducing the disability caused by leprosy.

2) **Treatment Regularity**

Regularity of treatment in patients with leprosy is a vital aspect of disease management. Despite the social stigma attached, disabilities caused by leprosy can often be prevented or well managed through regular and timely therapy [37, 72, 73]. Regularity of treatment not only ensures effective infection control, but also reduces the risk of nerve damage and permanent physical deformity [72, 74]. With regular monitoring and appropriate medical intervention, people with leprosy can minimize the impact of their disability, improve their quality of life, and reduce the risk of transmitting the disease to others.

3) **Self-Care**

Self-care for individuals with leprosy is crucial in managing their health condition, as the disease often results in significant disability. The self-care process not only involves medical treatment to control infection and prevent further nerve damage, but also includes aspects such as maintenance of personal hygiene, intensive skin care to prevent sores and infections, and physical exercises to maintain optimal mobility [75–77]. In addition, a holistic approach to self-care also takes into account the psychosocial aspects, by providing emotional and social support that is important for the psychological well-being of individuals living with this condition [44, 78]. As such, self-care is not just a medical act, but a comprehensive process that requires careful integration of various aspects of the health and well-being of the affected individual.

4) **Social Support**

People with leprosy often experience social stigmatization resulting in rejection and isolation from society. Social support from family, friends and the community is crucial in helping them overcome the physical, emotional and social challenges they face [44, 79]. Through such support, people with leprosy can feel accepted, valued, and motivated to undergo treatment and improve their quality of life [80, 81]. This includes practical help with daily activities, emotional support to overcome feelings of loneliness and depression, and social support in dealing with discrimination and stigma [82, 83]. Communities that provide positive social support also play a role in creating an inclusive environment and reducing stigma towards people with leprosy, so that they can live with more independence and dignity.

### Implications for practice

The practical implications of this review highlight the importance of healthcare providers adopting a comprehensive and integrated approach in managing patients with leprosy, with potential effects on clinical practice and public health policy. The identified factors, such as delayed diagnosis, suboptimal treatment adherence, and socioeconomic conditions, underscore the need for a more holistic approach to patient care. Based on these findings, healthcare providers are encouraged to adopt proactive preventive strategies, such as early detection and timely intervention, to prevent or minimize disability. Implementing comprehensive assessments that include both physical and social conditions should also be considered as part of patient management.

Furthermore, these findings support the importance of health education programs aimed at raising public awareness of leprosy. This may improve recognition of early signs and symptoms, facilitate earlier detection and treatment, and reduce stigma that often hinders care. Therefore, efforts to reduce stigma and discrimination should be integrated into public health strategies to improve treatment outcomes and the quality of life of patients with leprosy.

### Strengths and limitations

This review highlights that the factors contributing to disability in patients with leprosy are diverse and complex, and not easily classified, which may introduce bias in interpreting findings. The authors conducted a systematic thematic analysis, supported by collaborative discussions, to ensure that all relevant categories of predictors were considered. The study successfully identifies various clinical, social, and economic factors, while also highlighting gaps in the existing literature, particularly inconsistencies in demographic findings and limited integration across domains.

However, this study has several limitations. First, it included only studies published in certain languages, which may limit generalizability. Additionally, only published studies were included, which may introduce publication bias, and methodological variations across studies may affect data consistency. Second, a notable geographic concentration was identified: seven of the ten included studies were conducted in Indonesia. While this reflects Indonesia’s high leprosy burden and its status as the world’s third-largest endemic country, findings should be interpreted with caution when applying them to other regions with different epidemiological, cultural, and healthcare contexts. Readers outside Southeast Asia should note that contextual factors specific to Indonesia may limit the transferability of these results. Third, two of the ten included studies (Li et al., 2021 [29] and Tuturop et al., 2022 [31]) examined leprosy incidence rather than disability directly. These studies were retained because the factors they examined (socioeconomic conditions, healthcare access, treatment adherence) are contextually relevant to the risk of disability development, and their inclusion allows a broader mapping of the evidence landscape, consistent with the exploratory purpose of a scoping review. Nevertheless, readers should note that the evidence from these two studies is indirect with respect to the review’s primary focus on disability. Fourth, the JBI quality appraisal scores, while uniformly high (80–100%), were not used to exclude studies or to weight the synthesis, which is consistent with scoping review methodology. However, it should be noted that the high scores partly reflect the relatively small sample sizes of several studies, which may limit statistical power. Finally, this review was not prospectively registered in a protocol database, as discussed in the Methods section.

Future research is recommended to expand inclusion criteria, including unpublished studies and studies in a broader range of languages. The use of more standardized methodologies and longitudinal designs is also essential to obtain more consistent and in-depth findings.

## Conclusion

Disability in patients with leprosy is influenced by multiple factors categorized into demographic, internal, and external domains. Clinical factors, particularly diagnostic delay, multibacillary-type leprosy, and the number of nerves affected, appear more consistently associated with disability across the included studies, while demographic and socio-economic factors such as gender, education, and occupation currently show mixed and inconclusive evidence and should not yet be regarded as established predictors. Future research should prioritize longitudinal and integrative approaches to better understand these interactions and develop targeted interventions to improve treatment adherence and social support, ultimately reducing disability rates. Because seven of the ten included studies were conducted in Indonesia, these conclusions should be generalized to other leprosy-endemic settings with caution.

## Data Availability

All data generated or analysed during this study are included in this published article.
